# What Users Think about the Differences between Caffeine and Illicit/Prescription Stimulants for Cognitive Enhancement

**DOI:** 10.1371/journal.pone.0040047

**Published:** 2012-06-29

**Authors:** Andreas G. Franke, Klaus Lieb, Elisabeth Hildt

**Affiliations:** 1 Department of Psychiatry and Psychotherapy, University Medical Centre, Mainz, Germany; 2 Department of Philosophy, Johannes Gutenberg University, Mainz, Germany; The Scripps Research Institute, United States of America

## Abstract

Pharmacological cognitive enhancement (CE) is a topic of increasing public awareness. In the scientific literature on student use of CE as a study aid for academic performance enhancement, there are high prevalence rates regarding the use of caffeinated substances (coffee, caffeinated drinks, caffeine tablets) but remarkably lower prevalence rates regarding the use of illicit/prescription stimulants such as amphetamines or methylphenidate. While the literature considers the reasons and mechanisms for these different prevalence rates from a theoretical standpoint, it lacks empirical data to account for healthy students who use both, caffeine and illicit/prescription stimulants, exclusively for the purpose of CE. Therefore, we extensively interviewed a sample of 18 healthy university students reporting non-medical use of caffeine as well as illicit/prescription stimulants for the purpose of CE in a face-to-face setting about their opinions regarding differences in general and morally-relevant differences between caffeine and stimulant use for CE. 44% of all participants answered that there is a general difference between the use of caffeine and illicit/prescription stimulants for CE, 28% did not differentiate, 28% could not decide. Furthermore, 39% stated that there is a moral difference, 56% answered that there is no moral difference and one participant was not able to comment on moral aspects. Participants came to their judgements by applying three dimensions: medical, ethical and legal. Weighing the medical, ethical and legal aspects corresponded to the students' individual preferences of substances used for CE. However, their views only partly depicted evidence-based medical aspects and the ethical issues involved. This result shows the need for well-directed and differentiated information to prevent the potentially harmful use of illicit or prescription stimulants for CE.

## Introduction

Substance use for pharmacological cognitive enhancement (CE) by healthy subjects has received increasing attention during the last decade and is defined as the use of legal (i.e. caffeine) or illicit substances (i.e. illicit stimulants) as well as prescription drugs (e.g. methylphenidate, MPH) aiming at the enhancement of various cognitive functions (e.g. vigilance, concentration, memory) without medical need [1]–[5].

According to a recent metaanalysis by Wilens and colleagues, past-year prevalence rates of stimulant misuse in general ranged from 5% to 35% among students [6]. Whereas Wilens and colleagues did not differentiate prevalence rates with regard to the purpose of use (e.g. getting high, experimentation, concentration, academic performance enhancement) a more detailed analysis of the literature revealed that “concentration” and “study enhancement” is one of the most important intentions for stimulant misuse among students [7], [8]. Limited clinical effects for this purpose as well as potential side effects (e.g. tachycardia, agitation, jitteriness, gastro-intestinal symptoms) are described in numerous clinical trials and package inserts [2], [9], [10].

An online poll of the journal “*Nature*” depicts a lifetime prevalence rate of 20% of readers of this journal for stimulants, modafinil or beta blockers for the purpose of CE [11]. However, this was a highly biased non-random sample with the lack of representativeness. A preliminary study of our group has shown lifetime prevalence rates of 0.8% for prescription stimulants (MPH) and 2.9% for illicit stimulants (amphetamines (AMPH), cocaine, ecstasy) exclusively for CE purposes among German high school students [1].

Caffeine is the most widely-used wake promoting drug in the world with stimulant effects on the central nervous system (CNS). A random digit dialling survey in the US among 2,714 participants (25–74 years) showed that 78% are regular coffee drinkers, and only 15% had never drunk coffee [12]. Furthermore, a preliminary study of our group showed lifetime prevalence rates specifically for the purpose of CE in German university students of 54.9% for coffee, 30.5% for caffeinated/energy drinks and 10.7% for caffeine tablets [13]. In sum, prevalence rates of the consumption of caffeine exclusively for the purpose of CE are much higher than prevalence rates of stimulants [1], [6], [13].

In the interdisciplinary scientific literature, during the last few years there has been a vivid debate concerning CE. In this debate, scholars have been concerned with aspects such as efficacy, safety, cognitive liberty, autonomy, authenticity, identity, individuality, fairness, justice, equality of access, value of human effort, human nature and the “medicalization” of human life [3], [14]–[18].

Racine and Forlini identified three common paradigms that are used in order to discuss the non-medical use of prescription stimulants: the prescription drug abuse paradigm, the CE paradigm, and the lifestyle use of pharmaceuticals paradigm [19]. The first of these paradigms makes a clear difference between the non-medical use of prescription stimulants and the non-medical use of freely-available substances such as caffeine or Ginkgo biloba in that it harshly criticizes the non-medical use of prescription stimulants. In contrast, the other two paradigms, i.e. the CE paradigm and the lifestyle paradigm, do focus more on the divergent enhancing effects of the various substances, on the lifestyle context and on individual choice. In light of the two latter paradigms they do not draw a strict distinction between the various substances.

Generally speaking, there are two positions concerning the question about whether there is a moral difference between substances: On the one hand are scholars who argue that there is a moral difference, citing the following as evidence: detrimental medical aspects on health, negative implications concerning fairness and justice and issues related to individual identity, authenticity and medicalization of human life [18], [20], [21]. On the other hand are those who point out the similarities between caffeine and stimulant use. They argue that drug use for CE is in the same general category and in line with other kinds of improvements such as education, exercise, or meditation. Scholars in this camp stress individual autonomy and self-creation [3], [15], [22], [23].

To our knowledge, no one has conducted an investigation on the differences between the use of caffeine and stimulants ( =  illicit or prescription stimulants such as AMPH or MPH) for CE that is based on the views of persons who have used both substances for the purpose of CE.

## Methods

### Participants

22 university students were recruited by placards on all public bulletin boards throughout the Campus of the University of Mainz. As inclusion criteria we searched for healthy students who had used caffeine and (psycho-) stimulant drugs (AMPH, MPH) for the purpose of CE. Students with psychiatric disorders (e.g. attention-deficit hyperactivity disorder (ADHD), schizophrenia) with current physicians' prescriptions of psychoactive medication (e.g. Ritalin®) were excluded.

Potential participants were requested to contact us via email or telephone. Prior to participation, students gave written informed consent for the interview and sound recording. Participants received 30,- Euros as compensation for participating. The study was approved by the local Ethics Committee of the Landesärztekammer Rheinland-Pfalz (Medical Association Rheinland-Pfalz).

### Questionnaire and interviewing procedure

We developed an extensive semi-structured face-to-face interview guideline with consecutively numbered open and closed questions to gather information about the non-medical use of stimulants and caffeine for the purpose of CE. “Stimulants” are meant to be illicit stimulants (AMPH, cocaine, ecstasy) or prescription stimulants (MPH), but unlike caffeine which was explained to the participants prior to the interview. After asking socio-demographic questions (e.g. age, sex, study subject, grades, etc.) we asked a broad spectrum of questions concerning CE within the frame of a wider set of interviews among students (data about characteristics of students using AMPH and/or MPH for CE compared to a control group and a case study about characteristics of students using AMPH and MPH have been already published by Franke and colleagues (2012) and Hildt and colleagues (2011) [24], [25]). The two main questions we used to elicit participants' subjective opinions on the type of substance used for CE (caffeine vs. stimulant drugs) were: “Is there a difference between the use of caffeine and stimulants like AMPH or MPH for CE? Furthermore, is there a moral difference between the use of caffeine and stimulants like AMPH or MPH for CE? Please briefly explain why there is or why there is not a difference”.

One trained psychologist examined all participants for inclusion/exclusion criteria. The main goal of this pre-interview procedure was to ensure that all potential participants with psychiatric disorders (e.g. attention-deficit hyperactivity disorder (ADHD), schizophrenia) and/or current physicians' prescriptions of psychoactive medication were excluded from this study.

Prior to the interviewing process three interviewers were trained to interview participants following the written semi-structured interview guideline. Participants were always interviewed by two of these interviewers in a calm room. One interviewer asked the questions following the order of the semi-structured questionnaire, the other noted down participants' answers in order to add interview content in case of acoustic problems of sound recording. Records were transcribed by one third person who was not involved in the interview procedure.

### Coding and analysis

Interviews have been recorded, transcribed verbatim and analyzed systematically using a qualitative approach based on inductive category development [26]–[28].

Transcriptions were analyzed blindly by two independent raters. For reliability purposes, each rater came up with a set of three dimensions for the participants' arguments and justifications. There were no disagreements between the raters with respect to the choice of dimensions (medical, ethical and legal). Furthermore, both raters independently found subcategories for these three dimensions. Consensus was reached after discussion with regard to these subcategories in case of initial differences of opinion, thereby eliminating the need for a third rater.

## Results

In spite of eye-catching placards on numerous public bulletin boards around the entire campus of the University of Mainz (36,000 registered students), only approximately 30 students contacted us via email. After having planned the procedure and having applied the exclusion criteria, 22 interviews were carried out. Four interviews have not been analysed because of diagnosed ADHD, Pseudologia fantastica or technical reasons. All participants (100%, n = 18) had experiences using caffeine as well as prescription and/or illicit stimulants purposeful at least once exclusively for CE.

Participants were 25.8 years old (mean) and 2/3 of all participants were male; for further information about the characteristics of participants, see Table 1.

**Table 1:** Characteristics of participants.

Characteristics	Percentage/Number 100%, n = 18
Gender	66.7% male (n = 12) 33.3% female (n = 6)
Age (Mean ± SD)	25.8 years ± 2.88
Completed semesters (Mean ± SD)	7.35 semester ± 3.79
Department of - Humanities - natural sciences - economics	44.4% (n = 8) 33.3% (n = 6) 22.2% (n = 4)

Data are given as mean ± standard deviation (SD).

38.9% (n = 8) of all participants had used MPH, 77.8% (n = 14) answered that they had used AMPH, 22.2% (n = 4) had used both (MPH, AMPH) for CE. The prescription stimulant MPH only was used orally. In contrast, AMPH, which is an illicit drug in Germany, was administered intranasally by all AMPH users except one. The frequency of the stimulant use for CE varied widely; stimulant use ranged from one time only to several times, and even daily use (for a certain period of time, e.g. some weeks). Average age of first stimulant use for CE was 20.4 years, average age of first caffeine use for the purpose of CE was 16.2 years. All participants had already used caffeine (coffee, caffeinated drinks or caffeine tablets) for the particular intention of CE. Of these, all participants except two (88.9%, n = 16) had already used coffee at least once for CE, 55.6% (n = 10) caffeinated drinks and 55.6% (n = 10) caffeinated tablets at least once with this particular intention. Frequency of caffeine use for CE varied widely from once up to daily (for a certain period of time).

44.4% (n = 8) of all interviewed participants stated that there is a difference in general between the use of caffeine and stimulants for the purpose of CE. 27.8% (n = 5) answered that there is no difference at all, 27.8% (n = 5) were not able to decide whether there is a difference in general or not.

38.9% (n = 7) stated that there is a moral difference, 55.6% (n = 10) answered that there is no moral difference and one participant was not able to comment on moral aspects at all in spite of being asked for this explicitly.

After an analysis of interview transcriptions, we identified three dimensions by which participants argued and justified their points of view: ethical, medical and legal dimensions, including subcategories for the ethical and medical dimensions. Table 2 summarizes the main results.

**Table 2:** Dimensions of arguments and fitting subcategories for the use of illicit/prescription stimulants or caffeine for CE.

I. Medical Dimension	II. Ethical Dimension	III. Legal Dimension*
Efficacy	Self-harm and harm to others	
Desired effects vs. side effects	Modifications in behaviour and personality	
Predictability	Accessibility, fairness and justice	
Risk of Addiction	Individual decision-making and autonomy	
Type of effect and mechanism of action	Means-end-relation	
	Social conventions	

* Legal dimension cannot be subdivided.

### I. Medical Dimension

The medical dimension mentioned by all study participants can be subdivided into five different categories.

#### I.1 Efficacy

Nearly all (n = 16) participants based their opinions regarding the difference of substances by considering the respective substance's efficacy. The most frequently-stated medical argument of all participants was that the efficacy of stimulants is much more pronounced (“Caffeine was much less intense than stimulants”). They even tried to quantify the efficacy. (“Amphetamines have five-fold stronger effects than caffeine, I would say.”). On the one hand, participants praised the desirable efficacy of stimulants, but acknowledged the higher degree of side effects of stimulants and the better predictability of caffeine effects. Furthermore, they mentioned the higher degree of changed metabolism and the longer duration of effects of stimulants.

#### I.2 Desired effects vs. side effects

Ten participants argued that there is a notable difference between caffeine and stimulants regarding desired effects on the one hand and side effects on the other. The ratio of desired effects and side effects was very important for forming their opinion. Participants cited physical and mental side effects as important factors in their decision on using caffeine or stimulants. In particular, with regard to stimulant use, they mentioned an impairment to express oneself and “woolly thoughts”, as well as “modified ways of thinking, talking, behaving and different types of feelings”.

However, according to the answers obtained, stimulants had different effects in different people leading to enhanced or detrimental cognitive effects depending on the subject's type of use of the substance. To be more specific, participants' answers revealed more or less pro-cognitive effects regarding the use of stimulants if they used it only once or a couple of times (during a monthly or annual time frame). Using stimulants in a high frequency (e.g. daily, several times per week), the users find them to have rather detrimental cognitive effects.

Furthermore, participants thought about physical long-term damage, which was considered to be pronounced in the case of using stimulants. To some extent, participants argued that stimulants were more harmful for their body and brain than caffeine without giving closer explanations for this opinion. Remarkably, some argued that caffeine's desirable effects (wake promoting effects) and side effects (jitteriness) are well-known and that dosage was very easy to manage (counting cups of coffee) whereas imprecise dosage of stimulants could be dangerous and marginal inaccuracy could lead to severe “over-adrenalized”/“over-stimulating” side effects. Furthermore, according to them, caffeine had only wake-promoting effects while stimulants had “real” cognitive enhancing effects (e.g. on concentration). In addition, participants remarked that some time after intake the desired effects become transmuted into their opposite. (“One's state of mind after use is detrimental, and AMPH or Ritalin kills your inner life.”).

#### I.3 Predictability

Three participants differentiated between caffeine and stimulants in terms of predictability. They argued that types of effects and efficacy can be anticipated easily in the case of caffeine whereas the limit of predictability becomes a risk factor with stimulants. Dose-titration is particularly easy to manage by counting the number of cups of coffee. One important reason for the limited predictability of illicit stimulants was the risk of contamination (“Illicit stimulants can be cut…”). It was stated that it was necessary to buy black market stimulants; therefore predictability of the effect of black market substances was reduced given the possibility that they could have been cut.

#### I.4 Risk of Addiction

Five participants cited the risk of development of addiction regarding psychological dependence. They differentiated not only between stimulants and caffeine with a higher risk of addiction with stimulants, but also weighed the risk of addiction of prescription stimulants (Ritalin®) as being lower than the risk of illicit stimulants.

#### I.5 Type of effect and mechanism of action

Three students reported on different “clinical types” of effects. This ranged from wake promoting effects and “missing real cognitive enhancing effects” in case of caffeine to “real concentration enhancement” and “getting high” in the case of stimulants.

A few participants stated “scientific reasons” for differentiation between stimulants and caffeine and reflected about the influence on neurotransmitters. One participant even referred to the “clearance of reservoirs” in case of stimulant use without being able to specify involved neurotransmitters or neurotransmitter systems. Thus, he associated this clearance of reservoirs with strong effects directly after the use and feeling leached out and being excessively tired some hours/one day after having used stimulants. According to him, this induces re-use and leads to a vicious cycle (“… have the feeling of being far behind […], you have the demand for using stimulants again to get some power again because brain functions are reduced for a certain period of time […].)”.

### II. Ethical Dimension

Students differed in their opinions with regard to whether there is a moral difference between the use of caffeine and stimulants for CE. A typical answer of a participant who considered there to be no moral difference is: “Well, morally it doesn't make any difference. For me, either way, it's doping.”

Among the answers of those participants who stressed that there is a moral difference, the aspects mentioned can be subdivided in five different categories.

#### II.1 Self-harm and harm to others

Ten students stressed that in their view the misuse of stimulants, in particular of AMPH, involves a risk of harming one's body and organism which could have severely “negative implications”. However, sometimes students stopped without specifying these “negative implications”. By contrast, the participants consider it unlikely that the use of caffeine is very harmful to one's health. In this context, one student explicitly mentioned some kind of obligation to take care of one's body, which is needed throughout one's entire life.

According to another participant, there are no moral problems with using neuroenhancers as long as the user is able to estimate the consequences and no other person will be harmed.

#### II.2 Modifications in behaviour and personality

One of the participants considered that AMPH use can be problematic since its effects can include modifications in behaviour and in personality traits that may continue over several days. By contrast, he considered short-term problems due to caffeine use, such as sleeplessness, much more benign.

“When I can't sleep after spending three or four hours with coffee that's one thing, but I know people that change the way they act, the way they talk and the way they feel, after spending two or three nights without sleep, they sort of change and that's why [stimulants belong in] a different category for me.”

#### II.3 Accessibility, fairness and justice

Four students considered accessibility to be a relevant aspect from a moral point of view: Whereas caffeine is freely accessible to everybody, stimulants are not, which implies a lack of equal opportunities. Only one student explicitly mentioned fairness or justice. He described considerable advantages that accompany the use of stimulants (ability to revise and concentrate for a longer period of time) and considered it unfair to those who are not able to afford the drugs or who are not aware of them.

#### II.4 Individual decision-making and autonomy

Aspects related to individual decision-making and autonomy did not play a considerable role in the answers. Only one participant directly stressed individual autonomy, saying that it is up to each person to decide for herself which substance to take for CE purposes. For this reason the student opposes any kind of prohibition concerning drug use and argued for free access to enhancers.

#### II.5 Means-end-relation

Three participants argued that enhancers are just a welcome means to help them achieve their ends and that there is no moral difference whether they use coffee, energy drinks, Ritalin® or whatever else to achieve their ends.

“Well I have a target and I want to reach that target. And to reach it, I take the substance. I take it to reach my target faster or more effectively even though maybe it's just the good feeling that makes you work a bit more effectively. […] And then it doesn't matter what substance it is.”

The students mentioned ends such as achieving good scores at university and later obtaining a good job or performing better and increased productivity.

There were also some more critical opinions, however. One student said that achieving something without taking enhancing drugs gives him a better feeling.

Another one argued that the legitimacy of the type of substance to be used depends on the end a person pursues. He considered it legitimate for persons in special situations and with a high degree of responsibility to take stimulants for CE, such as medical doctors, pilots or those in the military.

#### II.6 Social conventions

Also the requirement to stick to social conventions played a role in three of the participants' answers. They said that the fact that stimulant use is not socially accepted plays an important role in determining the moral difference.

### III. Legal Dimension

A considerable number of students (n = 10) justified their opinions concerning differences between caffeine and stimulant use with arguments based on legal aspects. They stated that while stimulant use is illegal, caffeine is legal. It was mentioned that there would be a legal inhibition threshold. Illegality was a reason for keeping the use of stimulants secret: “The main problem is that [the use of stimulants] is illegal. Hence, you are treated like a criminal…”. The special German Narcotics Act (Betäubungsmittelgesetz, BtMG) was mentioned, too. However, three participants said clearly that they are not at all worried about law and that prohibition was not relevant for their decisions regarding whether to use caffeine or stimulants.

Several participants raised the point of legalization of prescription stimulants and illicit drugs. For most of them, the distinction legal – illegal was considered to be very important. Some spoke in favour of legalization and argued that if drug use were legal, it would then be much safer and information would be better. Others were against legalization of illicit drugs or were undecided concerning this question.

## Discussion

Participants' answers could be divided in medical, legal and ethical dimensions easily. The medical and legal dimension played a crucial role in the participants' answers to constitute their decision of using caffeine or stimulants for CE, whereas the ethical dimension was of limited importance for them.

Differences regarding effect(s) and efficacy of caffeine vs. stimulants were of notable relevance for the participants. However, caffeine has at least three established mechanisms of action: inhibition of cyclic nucleotide phosphodiesterase activity followed by an accumulation and potentialization of the effects of 3′5′-cyclic monophosphate (cAMP), blockade of adenosine receptors; mobilization of intracellular calcium) [2], [29]–[31] which are quite different from the mechanisms of action of stimulants (blockade of pre-synaptic norepinephrine and dopaminergic transporters leading to a reduction/loss of a negative feedback and thereby leading to an enhanced monoaminergic neurotransmission; this mechanism is strengthened by the vesicular release of dopamine by AMPH (not MPH)) [2], [32]–[34]. It is very difficult to agree or disagree with participants' statements that stimulants would change metabolism to a greater degree than caffeine. We can only assert that the mechanisms of action are quite different, therefore the respective mechanisms of action are not (directly) comparable. However, the aspect of the different mechanisms of action leads to the aspect of clinical efficacy, abuse and addiction.

Several participants stated that the (clinical) efficacy of stimulants is much higher than that of caffeine. However, clinical trials concerned with the efficacy of stimulants and caffeine that compare these two substances directly to each other in healthy subjects show that simple cognitive abilities (vigilance, reaction time, attentiveness) are increased by caffeine as well as AMPH with slightly stronger effects of 20 mg D-AMPH compared to 600 mg caffeine [2], [35], [36]. Furthermore, Wesensten and colleagues stated that the duration of the benefits vary in accordance with the different elimination rates of the substance [36]. However, there are no direct effects on higher cognitive abilities (e.g. memory) with either substance. Furthermore, AMPH has an additional mechanism of action compared to MPH leading to remarkable differences between AMPH and MPH [2]. According to the literature, stimulants and caffeine seem to be more or less equally effective regarding simple cognitive abilities, which are not comparable to complex cognitive abilities mentioned by the participants of our study (learning, understanding, etc.). The opinion of stronger effects of stimulants compared to caffeine may be caused by the expectation of stronger effects in cases of “illicit” drug use compared to legal and broadly-used substances like coffee. That different legal status may imply stronger (prescription and illicit stimulants) or weaker (caffeine) effects. In particular the restricted legal status of stimulants, which requires a specialized prescription, might lead to an overestimation of their effects and to an overestimation of one's own cognitive skills. Beyond that, in contrast to caffeine, the opinion of stronger effects of AMPH – and MPH to a smaller extent – could also be explained by indirect effects of stimulants on motivation and not just cognitive abilities [10].

Participants often stated a difference regarding the risk of addiction: students consider the abuse potential and the risk of addiction of stimulants to be undoubtedly higher than those of caffeine. Students' statements reflect what the literature shows. Prescription stimulants themselves have a certain risk of abuse and addiction [2], [37], [38]. Long-standing, these aspects lead to crucial controversy. Caffeine, however, causes “only” withdrawal symptoms in cases of abstinence (after long-term, high-dose use) [39], [40]. Furthermore, illicit stimulants (e.g. illicit AMPH, cocaine, ecstasy, etc.) are drugs of abuse and/or addiction. In our study, participants cite a higher abuse and addiction potential with regard to stimulants as compared to caffeine. It is important to consider how the participants administered their stimulants: All AMPH users except one have used stimulants intranasally which lead to pulsatile dopamine release. Because of this there is a remarkable higher abuse potential and risk of addiction than in case of oral use of stimulants.

Interestingly, in their answers, users often did not give considerable weight to the ethical dimension. This disregard stands in sharp contrast to interdisciplinary scientific literature in the CE debate, where ethical aspects of CE such as individual autonomy, cognitive liberty, authenticity, fairness, justice, pressure to perform and other social aspects play a crucial role [3], [4], [14]–[18], [20].

It seems that the users we interviewed focused on their individual situation and primarily did not account for conceptual aspects or broader implications of CE. Most of the users stressed the usefulness and the potential benefits and harms for themselves that go along with CE. What primarily mattered to them were efficacy, effects, and side-effects.

Only one participant explicitly mentioned individual autonomy as a main argument in favour of CE and in favour of some kind of liberal regulation concerning CE within society. However, a related argument on means and ends played a considerable role for several participants: CE is considered to be a means to better achieve one's ends. This argumentation relies on autonomy, too, albeit in a more indirect way than autonomy-based arguments that focus on free decision-making and the right to control one's brain chemistry. Choosing the means to achieve one's own goals is an aspect of autonomy, i.e. of the ideal to create and live one's life in a self-determined way. However, among the students interviewed, there is some lack of reflection on the adequateness of the means used. For some of the students, the end that they pursued even seemed to directly justify the means used.

It seems that most of the students we interviewed did not consider arguments that can be considered genuinely moral arguments, i.e. arguments that have to do with rights and obligations towards others. In fact, only one of the students we interviewed mentioned aspects related to fairness or justice, and only one student mentioned possible harm to others.

Genuine moral arguments would require a broader view which takes the interpersonal and social context into consideration. Instead, at least in their answers to the interview question, the students primarily focused on their individual situation and reflected on how to best achieve their ends. It seems that they did not thoroughly reflect on the context of their actions.

In the interviews, we did not give a definition of “moral” or “ethical”, so perhaps participants each had different notions of moral differences with regard to caffeine and stimulant use. Clearly, the answers we received represent folk morality, the views of those directly involved.

In contrast to this limited awareness of ethical issues regarding CE, the participants' argumentation focused heavily on the illegality of stimulant use. Students emphasized the fact that using stimulants in a non-medical context is something illegal, and this fact mattered to them. In Germany, ready-made AMPH-drugs (e.g. Adderall®) are illicit drugs and prescriptionable MPH (e.g. Ritalin®) falls under the German Narcotics Act (BtMG). We found that with regard to medical law, participants were well-informed.

When asked for moral reasons or moral arguments, a considerable number of students just mentioned illegality. For some, illegality is an important argument against the use of stimulants for CE purposes, while others argue for legalization of stimulant use for CE. It seems that illegality of stimulant use serves as a decisive argument that ends any further discussion. Moral arguments may play such a limited role given the illegality of stimulants.

Participants often began arguing amongst themselves about justifications for the use of caffeine or stimulants using divergent moral arguments, then came to the point of legal aspects and then promptly ended their discussion directly after naming legal aspects. Others named legal arguments earlier in their argumentation, then stopped and had to be asked by the interviewers to continue with their explanations. It seemed that the legal aspect was the “hardest” and most clear argument for them. The moral arguments seem to have been left to law-makers without recognizing the differences between those participants calling for liberalisation and those who did not.

The fact that illegality was so important in the users' evaluation of CE sheds some interesting light on calls for liberal guidelines or liberal policy approaches concerning CE such as the one put forward by Greely and colleagues [15]. Based on the results presented here, one may expect that liberalization would lead people to assume that there are no further relevant ethical issues with regard to CE.

After having discussed different aspects of students' answers given in interviews, let us now reflect on the status of the results obtained: To what extent are consumers' opinions regarding medical, legal and moral aspects of CE relevant?

The students told us just those aspects or ideas that came to their mind during the limited period of the interviews. This does not mean that their answers and aspects mentioned were the only ones they had ever considered regarding CE. Furthermore, we do not assume that they always responded candidly. To a certain degree, one might expect that answers are influenced by social expectations in interview situations. On the other hand, however, the atmosphere during the interviews was casual and we pointed out the anonymity of participants' responses so that we might reasonably expect to have obtained their genuine views.

It is important to stress that the interview responses do not tell us whether there is a moral difference between the use of stimulants or of caffeine. They do not give us any “objective” data concerning efficacy, effects and side effects either. Instead, they give us some ideas about the aspects that matter to the persons involved, of how they see the situation. This perspective helps to establish an empirically-informed basis for the discussion of medical, social and ethical implications of CE. Such an empirically-informed basis is an important presupposition for any kind of future policy recommendation or regulation concerning CE.

Beyond that, we have to admit that our findings have to be carefully interpreted and generalized. Interviews were carried out among a group of only 18 students who replied to placards on bulletin boards around the University campus which means that there is a selection bias. Furthermore, 2/3 of all interviewed participants are male and the age of the participants is relatively high for a university sample (mean: 25.8 years). Although these aspects limit the power of this study, we gained an initial insight about the reasons for the choice of the type of substance which is used for CE.

### Conclusion

Students using stimulants and caffeine for CE value medical, legal and ethical aspects to different extents. Less than half of the students see relevant differences between both substances. Medical and legal aspects play a major role, ethical reasons a minor role which seems to be overestimated in the literature. Weighing the medical, ethical and legal aspects corresponded to the individual preferences of substances used for CE. However, their views only partly depicted evidence-based medical aspects and the ethical issues involved. This result shows the need for well-directed and differentiated information to prevent the potentially harmful use of stimulants for CE.
